# Sharing data from clinical trials: the rationale for a controlled access approach

**DOI:** 10.1186/s13063-015-0604-6

**Published:** 2015-03-23

**Authors:** Matthew R Sydes, Anthony L Johnson, Sarah K Meredith, Mary Rauchenberger, Annabelle South, Mahesh KB Parmar

**Affiliations:** Medical Research Council Clinical Trials Unit at UCL, Aviation House, 125 Kingsway, London, WC2B 6NH UK

## Abstract

**Background:**

The move towards increased transparency around clinical trials is welcome. Much focus has been on under-reporting of trials and access to individual patient data to allow independent verification of findings. There are many other good reasons for data sharing from clinical trials. We describe some key issues in data sharing, including the challenges of open access to data. These include issues in consent and disclosure; risks in identification, including self-identification; risks in distorting data to prevent self-identification; and risks in analysis. These risks have led us to develop a controlled access policy, which safeguards the rights of patients entered in our trials, guards the intellectual property rights of the original researchers who designed the trial and collected the data, provides a barrier against unnecessary duplication, and ensures that researchers have the necessary resources and skills to analyse the data.

**Methods:**

We briefly discuss the practicalities of our current approach to data sharing, including ensuring that data are discoverable and how to deal with old studies. We describe data sharing activities at the MRC Clinical Trials Unit.

**Results:**

One hundred and three data sharing activities were logged from 2012 to 2014 from external and internal applicants. The motivations are varied, but none have been for replication of the primary results.

**Conclusions:**

For any request to share data, we note the important role of independent reviewers as well as reviewers who know the study well, and present some of the key questions that all reviewers should ask when deciding whether a request is reasonable. We consider the responsibilities of all parties. We highlight the potential for opportunity costs. Clinical trial data should be shared for reasonable requests but there are many practical issues that must be explicitly considered.

**Electronic supplementary material:**

The online version of this article (doi:10.1186/s13063-015-0604-6) contains supplementary material, which is available to authorized users.

## Background

The move towards increased transparency around clinical trials is welcome. Under-reporting hampers understanding of the benefits and harms of treatments. Reporting of trials and access to data are current issues, with much discussion around access to individual patient data (IPD) for independent verification of findings. However, there are other reasons for data release that stem from the ethical obligation to ensure optimal use of data collected in trials including:

- Independent verification of original results
- Placing the results of studies in a broader context: for example, individual patient data meta-analysis
- Secondary use of datasets; for example, using a trial as a convenience sample of high quality, prospectively-collected data to address issues different from the original research objectives
- Collaborating directly with other researchers where data need to be transferred to an alternative location for planned analyses: for example, pre-planned biological sub-studies or retrospective connection of individual outcomes to diagnostic samples
- Supporting evidence for planning new trials: for example, estimating event rate counts
- Developing new or improved statistical methodologies

The Medical Research Council Clinical Trials Unit at UCL (the ‘Unit’) has many years’ experience in designing, conducting, analysing and reporting clinical trials and other studies, including IPD meta-analyses and cohorts. Consequently, the Unit holds hundreds of datasets, extending back several decades, and is well versed in the issues associated with data sharing, both from the giver’s and recipient’s perspective.

Many organisations have recently published policy or guidance statements, or organised workshops on this issue, or submitted commentaries (Additional file 1: Table S1) [1-21]. The most comprehensive statement is the *Discussion Framework for Clinical Trial Data Sharing: Guiding Principles, Elements, and Activities* from the Institute of Medicine’s (IOM) *Sharing Clinical Trial Data: Maximizing Benefits, Minimizing Risk* [19] in the USA which followed a discussion process [8]. The final version of the European Medicines Agency (EMA) policy on data release, which will have regulatory impact throughout Europe, has recently been released [22]. The consultative engagement by the EMA with parties representing all relevant disciplines is noteworthy. The EMA has recognised that there are issues in sharing IPD and the published report focuses on access to Clinical Study Reports.

Our Unit fully supports the use of clinical study data for additional, ethical research with justified scientific objectives. However, we believe there are a number of problems with the completely open access approach advocated by some groups and journals. We describe these and other key issues we face in sharing data.

### Issues in consent and disclosure

We are mindful that people who consent to enter clinical trials are voluntarily subjecting themselves to experimentation and donating their personal clinical data to a team of medical researchers. They volunteer on the understanding that these researchers will conduct the trial responsibly, without unnecessary or illegitimate identification of the individuals involved. They may also allow their data to be passed to regulators for use in decisions about drug licensing. The protection of their data is described to the patient in the consent procedures when entering a trial. None of our clinical trials have asked for consent to disclose IPD data on the Internet. There are exceptions where explicit consent cannot be given directly by the patient and instead consent has to be provided by a third party: for example, trials in children or in adults who lack capacity through, for example, dementia or who are unconscious. We are unaware of published policy statements that address the issues of data disclosure in trials requiring assent.

### Risks in identification and self-identification

Many trial participants, especially those with chronic or rare diseases, are likely to be able to identify themselves in open access datasets from combinations of variables such as: age; sex; location; dates of consent, randomisation and follow-up appointments; and health parameters. Ideally, study doctors would discuss with participants all clinical information collected, but in the routine setting of busy hospital clinics, there is limited time and a host of blood test results that measure specific potential toxicities may be reduced to a simple ‘your blood tests were fine’ response. Self-identification in open access datasets and discovery of these test results may lead to concern and anxiety without the study doctor available to provide context and interpretation. Furthermore, adverse events may be technically classified as ‘serious’ according to standard regulatory definitions, but not be clinically ‘severe’ or regarded as ‘serious’ from the participant’s perspective. We regard disclosure of IPD outside the doctor-patient environment as potentially unethical and distressing, and possibly dangerous. Family and friends of a study participant or other inquisitive parties might also be able to identify them from open access data and learn sensitive, possibly stigmatising, information that the participant has chosen not to disclose.

### Risks in anonymisation and data distortion

Some people advocate for complete anonymisation or de-identification of clinical trials data. This reflects discussions in the context of ‘big data’. However, it is clear that current anonymisation methods are imperfect and that re-identification is possible [23].

Some advocate that data for open access can be distorted to prevent identification but distortion of key variables can disable researchers’ ability to replicate published results, thwarting a key aim of many advocates of broader data access. Distorting data does not completely de-couple it from patients in the trial. Participants could still use closest-match algorithms or even visually scan the dataset to identify the closest match to their own records. Some people might be distressed if fundamental characteristics like age and sex have been distorted and could even demand correction under data protection regulations. Distortion of continuous parameters may carry reported values across clinical thresholds which would normally trigger additional treatment: for example, blood pressure and anti-hypertensives or cholesterol and statins. This might cause concern to participants as to their management, or leave researchers reflecting inappropriately on the quality of the trial and its data. Therefore, distortion is not appropriate. Neither do we support attempts to distort data to anonymise or de-identify it, specifically to de-couple it from the original patients. We consider that such manipulation has not been addressed adequately in any of the published policy documents, and currently we do not regard it as feasible.

### Risks in analysis and dissemination

Unrestricted access to data could result in data dredging and a plethora of analyses that were never intended without the development of a sensible statistical analysis strategy. First, data dredging is likely to provide some false positive findings and lead to over-interpretation. Second, we are also concerned that unrestricted access to data could result in re-analysis of trials after their initial publication resulting in public disagreements about interpretation. Together these may lead participants to worry about what they have exposed themselves to, and raise public concerns about clinical trials in general. Sponsors have a duty of care to provide an adequate explanation to patients when there is controversy over the results.

Trialists set a high bar on who is qualified to analyse clinical trial data and pre-specification of key analyses is a pre-requisite of clinical trials. This standard must be maintained for subsequent uses of the data. Only a controlled access approach can achieve this.

### Opportunity costs

The time and effort required to prepare datasets for release should not be under-estimated. Receiving and processing applications, developing agreements and contracts, producing and transferring data, and responding to subsequent requests for clarification involves a broad range of functions across the Unit. In this resource-limited environment, there will inevitably be opportunity costs: data sharing activities may conflict with other demands on staff time, such as other ongoing research. An open access approach demands that the burdensome process of making all data ready for sharing is undertaken for all trials, regardless of the likelihood of future use of the data and whether support to prepare datasets is available. Core support is necessary from funding bodies to allow for the preparation of datasets. Even the activities in processing and considering applications requires large amounts of time. In the absence of core support, trials units may require applicants to provide funding to cover the necessary staff time. This is important, especially for older studies where the original grant has ended.

### Collaboration

A controlled access approach may bring opportunities for collaboration that an open access approach cannot. Trials unit representatives and other key trial team members should usually expect to be active collaborators in relevant projects built upon the trials work that they have undertaken, and not just passive providers of data.

## Methods

We reviewed data sharing activities from the trials and IPD meta-analyses at the MRC Clinical Trials Unit (CTU). For this purpose, we have defined a ‘data sharing activity’ as a request for IPD or summary data for one trial or meta-analysis beyond the original plans for that study, regardless of the outcome of the application for access to data. We have systematically logged activities since January 2012 and report to December 2014. Where possible, the motivation has been recorded. We use descriptive methods to summarize these activities.

## Results

We have logged 103 formal data sharing activities across 54 trials and IPD MAs in 2012, 2013 and 2014. A further 74 formal activities are logged on the system before this time, as are 4 informal activities for which formal applications are expected soon.

The release of data was approved in 80 of these 103 activities; 17 reviews are pending or ongoing, and 2 were abandoned by the applicants. Only four activities in this period were rejected. One request for data from three trials was rejected for scientific reasons and one was rejected because the trial data were not yet available. Seventy-one data sharing activities were led by external parties. Only four requests were for processed summary data, the majority being for IPD. Four activities in 2014 related to trials that recruited in the 1970s and 1980s, including one for which no data had ever previously been requested.

The most common motivation was methodology (41/103 activities), mostly internal to the unit. Other motivations were connecting trial data to biology samples (22), use in meta-analysis (13), other clinical projects (11) and other reasons (1). One activity was for re-analysis using alternative methods, relating to a tuberculosis (TB) trial that recruited in the 1970s. The motivation was not initially recorded on our data release forms and is unknown for 14 activities. None of the activities logged were to replicate the original analyses.

## Discussion

### MRC CTU at UCL approach

The approach we have developed to sharing data is in response to the considerations outlined above, in conjunction with our extensive experience of managing and sharing data from clinical trials and other studies across many specialties of medicine over a long time. We have chosen a controlled access approach whereby researchers make formal applications for data sharing. Key people, including the trial team and independent reviewers, review these. Our approach is based on a set of guiding principles that we believe should not be compromised:

- There must be a strong scientific argument or other legitimate rationale for the data to be used for the requested purpose
- No data can be released if this would compromise an ongoing trial
- Investigators who have invested time and effort into developing a trial should have a period of exclusivity in which to pursue their aims, before key trial data are made available to other researchers
- The resources required to process requests should not be under-estimated, particularly those needed to prepare data for release. Adequate resources must be available and the scientific aims of the study must justify the use of such resources
- All data exchange must comply with Information Governance and Data Protection Policies in all countries relevant to the disclosure
- All Unit trials must be ‘discoverable’; they are formally entered on recognised clinical trials registers, such as clinicaltrials.gov and controlled-trials.com, and listed on the Unit’s website [24]. Eligibility criteria for each study and a list of information collected in it are available (sometimes only on request). This combination of information is intended to prevent researchers from submitting requests that cannot be met, such as data that were never collected or populations that were not studied. Timelines to indicate when data might be made available may also be posted, subject to any necessary updating

### Processing data sharing applications

There are many necessary steps prior to any release of data. The Unit’s assessment process is staged and iterative, includes independent review, and may be terminated at any point. Our process, which applies equally to external and internal applicants, is detailed in the Additional file 1. Applicants are asked for information that allows reviewers to consider the: objectives; study design; qualifications and suitability; data required; samples required; ethical approval and consent requirements; planned outputs; authorship and publication policy; implications for the Unit; funding and resources needed and support available; and, timelines. A detailed protocol and statistical analysis plan are required along with other relevant documents: for example, grant applications, ethics approval.

Since applications for data sharing may be received long after a trial has closed and reported its primary results, the key study oversight committees should remain available for as long as feasible to consider them. Alternative procedures to provide late review should be decided before these key committees are disbanded. An approach the Unit has used is to ask the views of key ex-Trial Management Group (TMG) members, with independent review provided by another Trial Steering Committee (TSC) active in the same area of medicine. Tissue samples are a finite resource and careful discussion is required around access. Requests to access data from trials that have closed require specific approval from the Research Ethics Committee, and will consider issues in consent.

### The need for formal agreements

Successful external applications require a ‘data sharing agreement’, usually agreed at an institutional level. These agreements formalise key matters, including data transfer and storage, updates, publication, and the boundaries for further use, including, for example, that data can only be used for the specified purpose. Applicants must agree not to use the data to try to identify individual patients, unless this is a pre-specified purpose for record linkage: for example, to retrieve follow-up information on lost patients for an IPD meta-analysis. Our datasets may already include linked data from other sources: for example, National Health Service (NHS) Information Centre data on deaths. These are considered part of the dataset and can be included in data released by the Unit, subject to agreement and approval by the original source. Onward sharing, which is increasingly common around linked genetic samples, is restricted unless clearly specified and agreed (Additional file 1: Table S3).

### Preparation and transfer

To facilitate future data sharing, wherever appropriate and possible, we use standardised terms and definitions, standard collection and scoring tools and internationally agreed outcome measures [25]. The Unit takes particular care in releasing identifiable or sensitive data. Indeed, our data sets do not typically contain direct identifiers, but, if collected to facilitate data linkage, they are usually held separately from the main dataset and would not usually be included in released datasets. Indirect identifiers are usually removed prior to disclosure; age or year-of-birth may be used instead of date-of-birth, for example. Consideration is given to replacing ID numbers to break the link to our original dataset but de-identification through data distortion is not used.

Data may only be transferred by appropriately secure methods, after discussion with relevant experts in the Unit’s Data Management Systems and Information Services Group. Recipients must agree to store the data securely, according to Information Governance and Data Protection regulations.

### After data release

Recipients are asked to acknowledge receipt of data and to check immediately for problems. They must have appropriately qualified analysts, and may be asked to reproduce an already completed analysis to ensure the dataset is fully understood. A clear governance process is established to handle discrepancies between the original and replicated findings. Routes to discuss these findings and possible arbitration are established in the agreement if the purpose of data release is to investigate an alternative method of analysis that might lead to a different interpretation of a study’s findings.

Recipients are expected to publish their research promptly, according to their pre-specified plans. The Unit requires regular updates on projects involving released data for review by the study’s oversight committees. All parties are obliged to ensure that the data are used promptly.

### Other notable examples

GlaxoSmithKline (GSK) and partners have recently implemented the Clinical Study Data Request (CSDR) system [26]. This is also a controlled access model, although quite distinct from our own. With CDSR, requests for access to data are considered by, but not scientifically reviewed by, an independent panel [27]. Data are uploaded for trials at a time of each company’s choosing. After 1 year there were 1,200 studies available and 58 requests had been submitted [27]. The motives for the requests were not reported, but these analyses would be performed without the engagement of the original research teams and without collaborative opportunity.

## Conclusions

While strongly supporting the imperative to share data, we believe that there are a number of issues that require further consideration before open access to clinical trial data is possible and have therefore chosen to adopt a controlled access approach, where a reasonable request has a specific and justifiable scientific merit. This helps to safeguard the rights of patients who enter our trials, protect the intellectual property rights of the researchers who designed the trial and collected the data, and provide a barrier against unnecessary duplication. It also ensures that researchers have the necessary resources and capability to manipulate and analyse the data in accordance with their stated aims as specified in a statistical analysis plan. Funders and journals should withhold mandates for completely open access until these considerations have been adequately addressed. The current need to control access should never stand in the way of reasonable requests for data sharing.

## Additional file

Additional file 1:
**The additional file summarises the approach used for data sharing activities at MRC CTU, including some of the questions that reviews should consider.** It also considers some of the issues that researchers who have received data face when receiving requests to share the data further. The file also includes a list of organisations that have published interim or final policy or guidance statements or organized workshops.

## Abbreviations

ABPI: Association of the British Pharmaceutical Industry
BMJ: British Medical Journal
CSDR: Clinical Study Data Request
CTU: Clinical Trials Unit
DIA: Drugs Investigation Agency
EFPIA: European Federation of Pharmaceutical Industries and Associations
EMA: European Medicines Agency
FDA: Federal Drugs Agency
GSK: GlaxoSmithKline
IDMC: Independent Data Monitoring Committee
IOM: Institute of Medicine
IPD: individual patient data
MRC: Medical Research Council
NCRI: National Cancer Research Institute
NHS: National Health Service
PhRMA: Pharmaceutical Research and Manufacturers of America
PLoS: Public Library of Science
PSI: statisticians in the pharmaceutical industry
RCGP: Royal College of General Practitioners
SOP: standard operating procedure
TB: tuberculosis
TMG: Trial Management Group
TSC: Trial Steering Committee
UCL: University College London
UK: United Kingdom
USA: United States of America
