# Supplementary material for: Sharing data from clinical trials: the rationale for a controlled access approach
Source: Trials. 2015 Mar 23;16:104. doi: 10.1186/s13063-015-0604-6 (PMC4369803; doi:10.1186/s13063-015-0604-6)
Supplement: Additional file 1: — The additional file summarises the approach used for data sharing activities at MRC CTU, including some of the questions that reviews should consider. It also considers some of the issues that researchers who have received data face when receiving requests to share the data further. The file also includes a list of organisations that have published interim or final policy or guidance statements or organized workshops. [file 13063_2015_604_MOESM1_ESM.docx]

# SUPPLEMENT

## Processing Data Sharing Applications

The Unit’s assessment (**see below**) applies equally to external and internal applications, is staged and iterative, and may be terminated at any point. The stages apply to most studies, although our oversight structures vary, and therefore the consultant bodies, particularly in international trials or complex collaborations necessitate adaptation. Our template application form includes a summary diagram depicting our default process (**see below**).

Completing the application form is an iterative process. Applicants are asked for information that allows reviewers to consider their objectives; study design; qualifications and suitability; data required; samples required; ethical approval and consent requirements; planned outputs; authorship and publication policy; implications for the Unit; funding and resources needed and support available; and, timelines. A detailed protocol and statistical analysis plan is required along with other relevant documents e.g. grant applications, ethics approval. Reviewers each assess the application following the questions in **Supplement Table S2**. Reviewers’ comments are recorded on dedicated forms and summary feedback is provided regardless of outcome.

In the UK MRC oversight model for trials, the Trial Management Group (TMG) leads the trial and its members would review applications. The Trial Steering Committee (TSC) includes independent members, the chair amongst them, and they provide independent review. The TSC is the executive body for data release requests. The Independent Data Monitoring Committee (IDMC) with all independent members is the only body to see accumulating interim data and would be involved only if interim data were to be released.

## Stages of assessing applications to MRC CTU for access to data

- Discussion with Unit’s principal investigator, usually before formal application, to scope the science, practicality and resource requirements.
- Discussion of the detail of the intended proposal with the senior scientist at the Unit with overall responsibility for that research area.
- Review of the detail of the intended project by the Trial Management Group (or equivalent).
- Review by the Unit’s Scientific Strategy Group, especially if there are resource implications for the Unit, e.g. it would involve Unit staff in undertaking new analyses or collecting additional data.
- Review by the Trial Steering Committee (or equivalent), which includes independent members, to assess the impact on the study.
- Review by the Independent Data Monitoring Committee, if intermediate or immature data are requested.

## Data release SOP

Data release request forms, forms for comments on data release requests, and a clinical data disclosure agreement are available on the Unit’s website; the full version of the Standard Operating Procedure (SOP) (MRC_CTU_SOP_061) is available on request to the Unit Director.

**Table S1: Some organisations that have published interim or final policy or guidance statements or organised workshops**

| - Academy of Medical Sciences - UK[^1^](#_ENREF_1) - Association of the British Pharmaceutical Industry (ABPI) - UK[^2^](#_ENREF_2) - British Medical Journal (BMJ)[^3^](#_ENREF_3) - Cancer Research UK – UK[^4^](#_ENREF_4) - Drugs Investigation Agency (DIA) - USA[^17^](#_ENREF_17) - European Medicines Agency (EMA) - Europe[^5^](#_ENREF_5)^,^ [^22^](#_ENREF_22) - European Federation of Pharmaceutical Industries and Associations (EFPIA) – Europe[^6^](#_ENREF_6) - Federal Drugs Agency (FDA) - USA[^7^](#_ENREF_7) - GlaxoSmithKline (GSK)[^12^](#_ENREF_12) - Institute of Medicine (IOM) – USA[^8^](#_ENREF_8)^,^ [^19^](#_ENREF_19) - Medical Research Council (MRC) - UK[^9^](#_ENREF_9) - National Cancer Research Institute (NCRI) - UK[^10^](#_ENREF_10) - Nuffield Trust - UK[^14^](#_ENREF_14)^,^ [^15^](#_ENREF_15) - Pharmaceutical Research and Manufacturers of America (PhRMA) – International[^6^](#_ENREF_6) - Public Library of Science (PLoS) - USA[^11^](#_ENREF_11) - Royal College of General Practitioners (RCGP) – UK[^16^](#_ENREF_16) - Statisticians in the Pharmaceutical Industry (PSI) - International - Wellcome Trust – UK[^13^](#_ENREF_13) |
| --- |

Table S2: Key questions during review

| **Rationale, Merit, and Conduct of Project**   - Scientific merit of the proposal - Whether the trial data requested are suitable for answering the proposed research objectives - Biases that might be present e.g. is the study population likely to be representative of the applicants’ population of interest - Whether the original researchers or previous applicants already have plans to use the data in the way proposed by the applicants i.e. prevent duplication - Analyses are sufficiently well described to allow assessment that the methods in the proposal are fit for purpose - Whether the applicants have sufficient resources to complete their study - Motivation and suitability of the applicants, including whether the team (a) is suitably qualified to perform the analyses and (b) has the facility to handle the data. |
| --- |
| **Protection of Participants**   - How would confidentiality be maintained? - Does the original documented patient consent cover the proposal to release their data? - Is approval by a Research Ethics Committee required? |
| **Protection of the Integrity of the Original Trial**   - Would the proposed use of data jeopardise the conduct or results of the trial from which the data are derived? If so, the proposal is unlikely to be supported at this time. - Is there duplication? Have these data already been requested for this purpose? - Are there implications for the future conduct or interpretation of the trial |
| **Resource Implications**   - What resources would be required at the Unit to help investigators to understand the data, prepare the dataset, transfer the dataset, and perform the specified analyses. - Are these resources available to or from the Unit? - If not, could they be made available? - What is the opportunity cost? [Needs explanation]. - Are Unit staff considered providers of data or full collaborators? - Are raw or summary data required? - What version of the trial dataset is required? - Do new data need to be collected? If so this will need approval by a Research Ethics Committee. |

**Table S3: Issues in onward sharing of data**

| Data from a Unit trial or study that are released to others may be subject to further requests for sharing. For example, a trial dataset may be released to a Researcher A for inclusion in a meta-analysis, and another Researcher B might then request Researcher A for access to the combined dataset for further analysis. Such onward sharing is strictly prohibited without the explicit consent of the Unit. Generally, onward sharing to Researcher B is likely to be straightforward if Researcher B wishes to use the data in a project that has a purpose in line with the original release to Researcher A. If Researcher B wishes to use the data for a purpose that was not covered by the original data release agreement, Researcher B must contact the Unit directly. Should Researcher A ever release data to Researcher B, a formal agreement must be developed by the researchers’ institutions which sets out terms of use that are at least as stringent as those in the original agreement involving the Unit. The Unit must be informed of the intended release before it takes place. |
| --- |
